# The Role of Genetic Polymorphisms in Diabetic Retinopathy: Narrative Review

**DOI:** 10.3390/ijms242115865

**Published:** 2023-11-01

**Authors:** Edyta Sienkiewicz-Szłapka, Ewa Fiedorowicz, Angelika Król-Grzymała, Natalia Kordulewska, Dominika Rozmus, Anna Cieślińska, Andrzej Grzybowski

**Affiliations:** 1 Department of Biochemistry, Faculty of Biology and Biotechnology, University of Warmia and Mazury, 10-719 Olsztyn, Poland; edyta.sienkiewicz@uwm.edu.pl (E.S.-S.); ewa.kuzbida@uwm.edu.pl (E.F.); angelika.krol@uwm.edu.pl (A.K.-G.); natalia.smulska@uwm.edu.pl (N.K.); dominika.rozmus@uwm.edu.pl (D.R.); 2 Institute for Research in Ophthalmology, Foundation for Ophthalmology Development, Gorczyczewskiego 2/3, 61-553 Poznań, Poland; ae.grzybowski@gmail.com

**Keywords:** diabetic retinopathy, polymorphism, chromosome, diabetes

## Abstract

Diabetic retinopathy (DR) is renowned as a leading cause of visual loss in working-age populations with its etiopathology influenced by the disturbance of biochemical metabolic pathways and genetic factors, including gene polymorphism. Metabolic pathways considered to have an impact on the development of the disease, as well as genes and polymorphisms that can affect the gene expression, modify the quantity and quality of the encoded product (protein), and significantly alter the metabolic pathway and its control, and thus cause changes in the functioning of metabolic pathways. In this article, the screening of chromosomes and the most important genes involved in the etiology of diabetic retinopathy is presented. The common databases with manuscripts published from January 2000 to June 2023 have been taken into consideration and chosen. This article indicates the role of specific genes in the development of diabetic retinopathy, as well as polymorphic changes within the indicated genes that may have an impact on exacerbating the symptoms of the disease. The collected data will allow for a broader look at the disease and help to select candidate genes that can become markers of the disease.

## 1. Introduction

**Figure 1:**
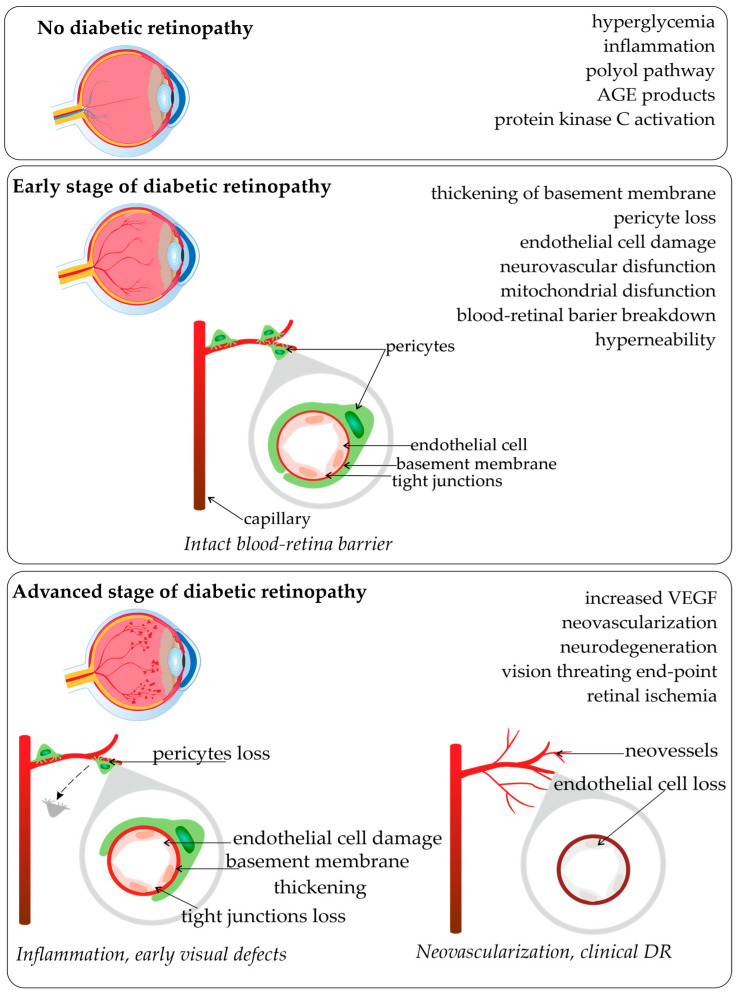
Pathological vascular changes in diabetic retinopathy (based on [2,6,7,8,9]; made with SciPainter Beta ver. 1).

Diabetic retinopathy (DR) is renowned as a leading cause of visual loss in working-age populations [1,2]. For patients with diabetes mellitus type 2 (DM2) who survive for over 20 years with the disease, 77% of them also suffer from diabetic retinopathy [3,4]. The major risk factors for DR development and progression include the duration of diabetes, high levels of glucose and lipids in the blood, hypertension, as well as pathogenetic pathways and genetic factors [3,5]. Biochemical factors with genetic influences that were proposed as modulators of the pathogenesis of retinopathy and disturbance of metabolic pathways were associated with the accumulation of sorbitol, advanced glycation end-products (AGE), oxidative stress, protein kinase C activation, inflammation, and upregulation of the renin-angiotensin system, vascular endothelial growth factor (VEGF), and aldose reductase [2,6,7]. Based on epidemiological studies, it is known that the development of retinopathy is related to the glycemic index and the duration of diabetes [7]. Diabetic hyperglycemia influences endothelial cell dysfunction, endothelial cell death by apoptosis, and consequent retinal capillary loss seen in diabetic retinopathy. The scheme of common pathological vascular changes in DR development, and the stages of disease are present in Figure 1.

As diabetic retinopathy occurs in the background of DM, genetic factors playing a major role in the etiology of DM have long been appreciated because of ethnic differences in frequency, increased familial aggregation, and a markedly higher concordance in monozygotic vs. dizygotic twins [10]. While glycemic control is critical in the development of retinopathy, the identification and characterization of genetic factors can also improve the means of preventing and treating of this destructive disease [11]. Several pathways and processes have been proposed to play an important role in the pathogenesis of DR, which led to the testing of a number of hypothesized candidate genes. Metabolic pathways considered to have an impact on the development of the disease were listed, as well as genes and polymorphisms involved in the process [10,12]. DR is seen as a polygenic disorder [13], and genetic factors account for 25–50% of the risk of developing DR [14]. Genetic polymorphisms (Single Nucleotide Polymorphisms (SNPs)) can affect the gene expression, modify the quantity and quality of the encoded product (protein), and significantly alter the metabolic pathway and its control, and thus cause changes in the functioning of metabolic pathways [15,16,17,18]. Statistically significant associations between various polymorphisms and DR were reported in many individual studies; however, many of the results are conflicting [19]. Identifying genes is more challenging because of the complexity of multifactorial, polygenic and environmental influences on disease development [12].

This article links the screening of chromosomes, the most important genes involved in the etiology of diabetic retinopathy, and the role of polymorphisms in the development of disease.

**Figure 2:**
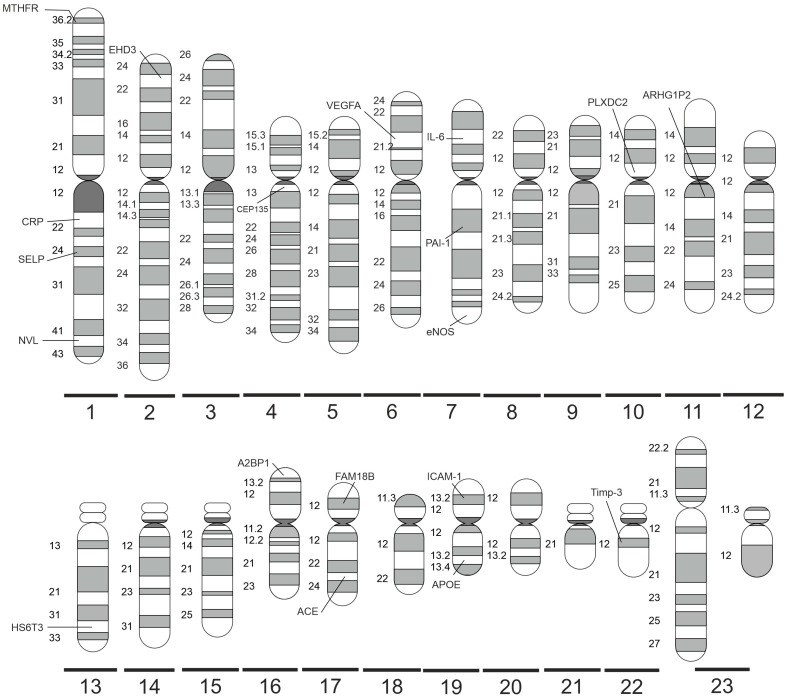
Significant DR-related genes distributed on specific human chromosomes (made with SciPainter Beta ver. 1).

Figure 2 shows a summary of the significant polymorphisms distributed on specific chromosomes, and their detailed description is provided below. 

## 2. Materials and Methods

### Literature Search Strategy

Here, information on gene polymorphism data in diabetic retinopathy is presented according to the chromosomes. The data were organized according to chromosome location as a way to more conveniently and orderly present the gene polymorphisms.

Databases: PubMed, Web of Science, Cochrane Library and Google Scholar were searched for articles published from January 2000 to June 2023. In the Google Scholar database, the research sequence used was: diabetic retinopathy human “chromosome [number]” “gene polymorphisms” -animal -mouse -rat -chicken -ape -monkey -rabbit -pig -cat -dog -infant -cancer -tumor -neoplasm -glaucoma -trauma -infection. The search was repeated in case of a small number of records using sentences: retinopathy human “chromosome [number]” “gene polymorphisms”.

In PubMed, Web of Science, and Cochrane Library, the research words were: diabetic retinopathy, human chromosome [number], and gene polymorphisms. Articles published within the last 23 years were selected. The reference lists of articles were identified by this search strategy and selected only those relevant (few articles < 2000 year). Additionally, during the research, each time a given gene was found, it was also checked for retinopathy.

All available publications in English and those having English abstracts were reviewed. In the end, only original studies that described the role of polymorphisms in retinopathy were included. Publications were excluded: (1) if they were duplicated; (2) if were published prior to 2000; (3) had unclear methodology or obtained data; (4) when systematic reviews in which all relevant studies were captured in another more recent review.

**Figure 3:**
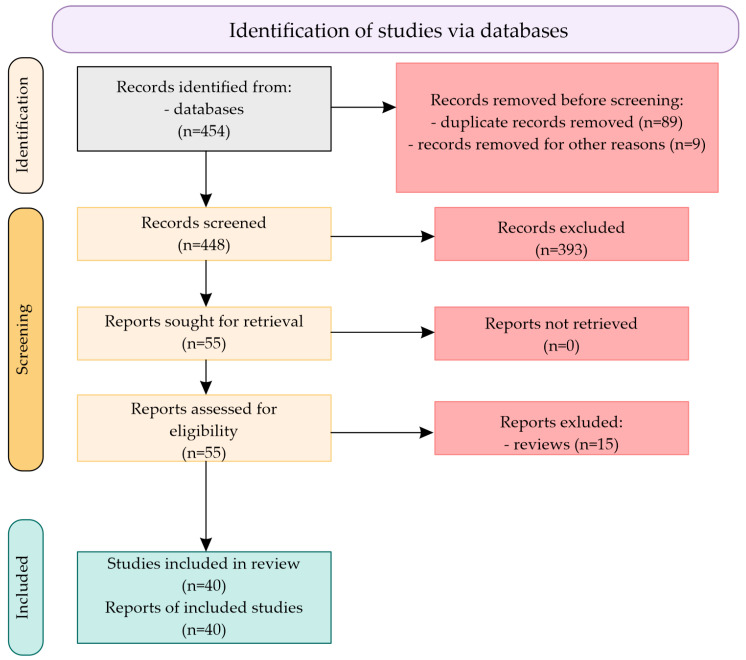
Scheme of literature searching method.

Each analyzed SNP was also confirmed in the dbSNP of NIH database [www.ncbi.nlm.nih.gov/snp]. A total of 454 citations were identified in the literature search, screening of titles and abstracts excluded 393 manuscripts, and 55 potentially relevant reports from the electronic search were retrieved for full-text review. Of these potentially relevant articles, 15 publications were excluded for various reasons, and 40 publications met the inclusion criteria and were included in this report. Figure 3 shows the scheme of literature searching method.

## 3. Results and Discussion

The results of the most important genes involved in retinopathy etiology are presented in order of chromosome numbers, with a brief characteristics of genes, action, and role of polymorphisms in reference to disease.

### 3.1. Chromosome 1

Coded by *SELP* gene, P-selectin (GMP-140, PADGEM, CD62P) is a cell adhesion molecule (CAM) present on the surface of activated vascular endothelial cells and thrombocytes. This carbohydrate-binding protein is a highly glycosylated membrane protein, the structure of which consists of five parts: an N-terminal C-type lectin domain, followed by an epidermal growth factor (EGF)-like domain, nine consensus repeats (~60 amino acids), a transmembrane domain, and a short cytoplasmic tail [20,21,22]. Newly synthesized P-selectin is stored in Weibel-Palade bodies of endothelial cells or in α granules of platelets. Upon stimulation with inflammatory mediators such as thrombin, histamine, or components of the complement system, P-selectin is rapidly redistributed to the cell surface where it is present mainly in the form of monomers or forms dimers or oligomers as an effect of transmembrane domain interaction. The appearance of P-selectin on the endothelial cell surface is very fast and reaches its peak 3–4 min following stimulation and then declines to basal level within 30 min [20,22]. The lectin domain of P-selectin interacts reversibly, mainly with P-selectin glycoprotein ligand-1 (PSGL-1), a transmembrane homodimeric sialomucin on leukocytes, lymph nodes vessels, and endothelium. These interactions support hemostasis by mediating platelet-leukocyte interactions at the place of damaged blood vessels and are essential in the rolling of leukocytes on activated epithelial cells, as well as for their movement to the inflammatory site [20,23]. Activation of PSGL-1 also stimulates the secretion of IL-8 by endothelial cells, which induces a series of physiological responses required for cell migration and phagocytosis and strongly promotes angiogenesis [21]. Once expressed on the cell surface, platelet P-selectin remains there until it is shed into the plasma (ectodomain, sPsel), whereas endothelial P-selectin is rapidly internalized in clathrin-coated pits. In humans, a minor portion of sPsel is derived from alternative mRNA splicing that removes the exon 14 encoding the transmembrane domain, while the primary contributors to the formation of sPsel in the physiological state seem to be platelets (baseline concentrations) [20,22]. In normal subjects, sPsel concentration is gender-dependent (higher in males) and ranges from 15 to 100 ng/mL [22]. Elevated levels are identified in cardiovascular diseases (myocardial infarction, thrombosis, stroke, and atherosclerosis) and occur already in early stages of DM1 and DM2 without vascular complications, insulin resistance, and IFG [22,23]. It is considered that diabetes-induced expression of adhesion molecules, such as P-selectin and ICAM-1, causes increased leukocyte–endothelial cell adhesion, leukocytes entrapping in the retinal capillaries (leukostasis), localized production of reactive oxygen species (ROS), blood–retinal barrier breakdown, capillary nonperfusion (formation of microthrombi), and the vascular damage (microangiopathy) [21,23]. It should be noted, however, that results on mouse models indicate that circulating monomeric sPsel is not prothrombotic or proinflammatory unless it undergoes di- or oligomerization [22]. Additionally, it is suggested that, provoked by P-selectin, leukocytes could facilitate proliferative damage of vasculature, thus the potential function of P-selectin is suspected during both stages of diabetic retinopathy, non-proliferative (NPDR) and proliferative (PDR) [24]. The genetic variants of this low-grade inflammation biomarker in association with diabetic retinopathy have been investigated in several projects (Table 1). In a large international study [25] in patients with DM2, the three SNPs in the *SELP* gene (rs6128, rs6133, and rs3917779) were found to be associated with DR but only in the European American population, and not in other ethnic groups. Whereas Penman et al. [26], in ethnically homogeneous African American groups of DM2 and IFG patients, showed an association between the rs6128 genotype, plasma P-selectin levels, and diabetic retinopathy. The genotype and allele frequencies of these three polymorphisms were analyzed also in PDR patients in Yazd, Iran [24]. In this population, rs6128 and rs6133 polymorphisms have not been shown to be associated with the proliferative stage of diabetic retinopathy. This is in contrast to the rs3917779, which showed significant differences in the distribution of genotypes and a strong association with PDR (CC genotype). According to the NCBI database of genetic variation (dbSNP) search results (15 July 2023) among three of these polymorphisms, only rs6133 (exon 12) could be potentially functional. The rs3917779 is an intronic variation (intron 10), but it is located in a binding site for transcriptional repressor CTCF and could be involved in the negative regulation of transcription, genomic imprinting, or alternative splicing of P-selectin [24]. Finally, rs6228 is silent genetic variation, but it is localized in exon 14, which is eliminated in alternative splicing and results in the production of soluble P-selectin molecules. Thus, it could be indirectly related to different levels of sPsel in plasma [27].

The *MTHFR* gene encodes a rate-limiting enzyme 5,10-methylenetetrahydrofolate reductase (EC 1.5.1.20) involved in the metabolism of S-adenosyl methionine (SAM), catalyzing the reduction of 5,10-methylenetetrahydrofolate to 5-methyltetrahydrofolate (5-MTHF), which in turn is necessary for the conversion of homocysteine to methionine (in a reaction catalyzed by methionine synthase (MTR)). Several polymorphisms in this gene have been reported, but the most studied are rs1801133 (C677T, exon 5, resulting in Val to Ala substitution at 222 amino acid chain position, N-terminal catalytic domain) and rs1801131 (A1298C, exon 8, resulting in Ala to Glu substitution at 429 amino acid chain position, C-terminal regulatory domain) [28,29]. The common genotypes CC at 677 position and AA at 1298 position are completely functional, whereas missense polymorphic variants result in the synthesis of a less efficient enzyme. Heterozygous allelic combination 677 CT provides about 65% of MTHFR activity, and 677 TT genotype variant gives less than 30% of enzyme activity in the processing of folic acid. A less significant decrease in MTHFR activity is noted in heterozygous allelic combination at the 1298 AC (estimated for about 80%) and for variant 1298 CC it is about 60%. Double heterozygotes have an additional loss of enzyme activity, 50–60% of normal. However, double homozygotes are considered very rare. Therefore, the presence of the 677 TT enzyme isoform is mainly associated with high levels of homocysteine (Hcy), an amino acid that is suspected to serve as a proinflammatory factor, inducing endothelial dysfunction and arterial stiffness, increasing the risk of cerebrovascular and cardiovascular diseases, including atherosclerosis, as well as retinopathy in DM1 and DM2 [28,30]. Homozygous TT subjects are predisposed to hyperhomocysteinemia, particularly when their folate status is low [31]. The increased supply of folic acid in the diet of the 677 T and 1298 C alleles carriers is considered to be a protective factor providing a better supply of 5-methyltetrahydrofolate (5-MTHF) to subjects with naturally occurring lower MTHFR activity. However, to decrease the level of Hcy, a methylated derivative of vitamin B_12_ is also required, which is necessary for the use of 5-MTHF by the next step in the SAM cycle (MTR enzyme cofactor). Vitamin B_12_ deficiency results in a functional deficiency of vitamin B_9_ active form and intensifies the negative predisposition of the 677 T and 1298 C alleles carriers to generate higher Hcy levels, and—what could be even more important—to the disturbances of methylation processes (including DNA methylation) due to reduced SAM biosynthesis. The lowered status of genomic DNA methylation in individuals heterozygous and homozygous for both polymorphisms (independently and concurrently to both loci), especially 677 TT, has been proved by Castro et al. [32]. This aspect expands the consideration of associations between these two polymorphisms and the development of diabetic retinopathy, including the possible involvement of epigenetic mechanisms. Moreover, in vitro data and some clinical study results suggest that disturbances to the SAM cycle in these conditions could be enhanced by hyperinsulinemia and insulin resistance, as insulin was found to decrease the expression of MTHFR in cells [33]. It should be noted here also that the retina is one of the most responsive targets of insulin action, where the expression of insulin receptors is higher than in the liver or muscles [34]. At the turn of the 20th and 21st centuries, numerous studies were performed to estimate the relationship between the *MTHFR* C677T polymorphism (rs1801133) and DR, but the results were inconclusive, probably because of the ethnic differences. A comprehensive meta-analysis of data collected between 1996 and 2016 was made by Luo et al. [30]. They estimated the results for two populations of patients: Asian and Non-Asian (Caucasian and African American), 1747 diabetic retinopathy cases, and 3146 controls (healthy and diabetic free of diabetic retinopathy). From the stratification analysis by ethnicity and DM type, they stated that the rs1801133 polymorphism was significantly associated with DR risk in the DM2 group and in the Asian populations (regardless of DM type) (Table 1). A meta-analysis performed on studies of A1298C polymorphism association with DR has been made by Garcia-Hernandez et al. [35] (Table 1). Interestingly, out of 15 publications (1998–2018) related to A1298C polymorphism association with DR, and due to the availability and quality of data, only 4 were able to be selected for meta-analysis. All the studies involved included patient populations from North Africa and the Middle East. It was found that the A1298C *MTHFR* polymorphism could increase the risk of developing diabetic retinopathy in Arabian countries, but only in the dominant genetic model. However, as the authors emphasized, this association was not very representative due to significant sample heterogeneity, small general sample size, or possible environmental factors affecting the data (like folate consumption or diabetes management). In general, meta-analysis points out mainly the lack of broader studies covering the A1298C polymorphism in DR. Recently, both SNPs were investigated in one meta-GWAS (Genome-Wide Association Study) research evaluating their association with DR risk and progression in DM2 diabetic patients in Pakistan [36]. The obtained results showed no linkage of the rs1801131 (A1298C) polymorphism neither to the risk of DM2 nor to any type of diabetic retinopathy (NPDR or PDR). Whereas quite surprising results were obtained for rs1801133 (C677T), as in both groups (healthy and diabetic) the TT genotype was very frequent (63% and 76%, respectively), and the T allele showed a significant protective effect against DM2, although not against the development of diabetic retinopathy (Table 1). As it is postulated, the discrepancies found in the results of the studies discussed above could arise from the genetic differences of ethnic groups, small sizes of study groups, or imperfections and diversity of study protocols. However, some other factors, such as the type of diet, especially in terms of vitamins B_9_ and B_12_ supply, may also be essential.

The *NVL* gene encodes nuclear VCP-like protein 2 (nuclear valosin-containing protein-like 2, NVL2), which is a member of the AAA-ATPase family. NVL2 shows specific localization to the nucleolus and exhibits a high degree of structural similarity to the ubiquitin-selective chaperone VCP/p97. As an ATP-ase and RNA-dependent protein, it is involved in ribosome biogenesis and pre-rRNA processing. It is also required for telomerase assembly and the regulation of its activity. The *NVL* gene is widely expressed in vivo with the highest expression in the retina. The first report on the possible association of *NVL* gene variation with the development of diabetic retinopathy was based on the results of the largest Genome-Wide Association Studies dedicated to DR (over 43,000 subjects, meta-analysis for multiethnic replication cohorts) [37]. In this study, the rs142293996 (22nd intron, allele C) has been found to be the most significant genetic variable for European patients with extreme DR, with the same direction in replication cohorts, but without genome-wide significance. Furthermore, using liability threshold (LT) modeling with diabetes duration and glycemic control as parameters, and by testing if the association was a significant cis-expression quantitative trait locus (eQTL) in the Genotype-Tissue Expression (GTEx) Project release v7, authors have found an rs142293996 association with the *CAPN2* gene, so they concluded its possible association with serum alpha-carotene levels as a potential functional role in DR development.

C-reactive protein (CRP), the product of *CRP* gene, is a ring-shaped pentameric protein, and a very sensitive plasma marker of inflammation (a classical acute phase reactant) produced rapidly in the liver in response to cytokine-mediated action triggered by injury or infection (mainly to IL-6 secreted by macrophages and lymphocytes T). Its biological function is to activate the complement system [38]. CRP is involved in endothelial dysfunction and angiogenesis, stimulates leukocytes—endothelium interactions, decreases endothelial nitric oxide, and impairs the function of endothelial progenitor cells [39]. Some studies have reported that higher CRP could be associated with the prevalence of DR [40], others have a contrary opinion that patients with higher levels of CRP are less likely to have DR [41]. This could be associated with different variants of *CRP* gene, like those located downstream of *CRP* rs2808629 [42]. One study performed in Shanghai showed that the G allele of rs2808629 was significantly associated with increased susceptibility to retinopathy in patients with DM2 for over 10 years [39]. Whether this effect is common or rather restricted to the Chinese population needs to be investigated. In parallel, more research is required to establish whether elevated CRP levels (if any) could be related to the early events of retinopathy (a triggering factor, in accordance with the low-grade inflammation hypothesis) or rather to the development of microvascular complications. Table 1 presents the most important polymorphisms related to retinopathy found on chromosome 1.

**Table 1:** Polymorphisms of genes related to diabetic retinopathy on chromosome 1.

Gene	Polymorphism or rs ID Number	No. of Participants	Results	References
*SELP*	rs6128	DM n = 2691 DNR n = 1032, DR n = 222 (EA) DNR n = 552, DR n = 271 (AfA) DNR n = 54, DR n = 25 (AsA) DNR n = 151, DR n = 80 (HA)	The SNPs in *SELP* gene were associated with DR only in the European American population. The strongest association was found to rs6128 (OR = 0.43, *p* = 0.0001), whereas to rs6133, and rs3917779 the odds ratios were: OR = 0.38, *p* = 0.0004 and OR = 0.39, *p* = 0.0006, respectively. Association was also not significant in independent Asian and Caucasian (Europe) cohorts.	[25]
	rs6133			
	rs3917779			
*SELP*	rs6128	DM2 n = 629 IFG n = 266 (AfA)	The rs6128 genotypes distribution in three subgroups (healthy, IFG, DM2) had no significant differences, but participants without retinopathy were more likely to be minor allele homozygotes (TT; 5.7%) than those with retinopathy (*p* = 0.03). The rs6128 minor allele homozygotes had lower mean P-selectin plasma levels than major allele carriers (25.8 ng/mL vs. 34.5 ng/mL; *p* = 0.046). There were no significant associations with either retinopathy or P-selectin levels for rs6133 and rs3917779.	[26]
	rs6133			
	rs3917779			
*SELP*	rs6128	DM2 n = 110 DNR n = 55 PDR n = 55 (Yazd, Iran)	rs6128 and rs6133 variations were not associated with PDR; significant different distributions of rs3917779 alleles and genotypes were found: allele C frequency was higher in PDR group (OR = 42.9, *p* < 0.001); CC genotype had a frequency of 0.96 and was strongly associated with PDR (*p* < 0.0001); in the DNR group, frequency of TT genotype was 0.62 (*p* < 0.0001); no heterozygotes were identified in either group.	[24]
	rs6133			
	rs3917779			
*MTHFR*	rs1801133 (C677T)	DR n = 1747 (n = 1141 As, n = 606 NAs) CTR (DNR and H) n = 3146 (n = 1589 As, n = 1557 NAs)	Analysis in the overall group in the genetic model CT vs. CC revealed OR = 1.46, *p* < 0.01, and in the model TT vs. CC revealed OR = 2.45, *p* < 0.01 (Rmo OR = 1.67, *p* < 0.01, and Dmo OR = 1.71, *p* < 0.01). From stratification analysis by ethnicity: in Asian populations CT vs. CC revealed OR = 1.71, *p* < 0.01, and TT vs. CC revealed OR = 2.97, *p* = 0.02 (Rmo OR = 2.16, *p* = 0.11, and Dmo OR = 1.98, *p* < 0.01); in non-Asian populations CT vs. CC revealed OR = 1.15, *p* = 0.38, and TT vs. CC revealed OR = 1.33, *p* = 0.04 (Rmo OR = 1.24, *p* = 0.05, and Dmo OR = 1.18, *p* = 0.21). Subgroup analysis by DM type: in DM2 group CT vs. CC revealed OR = 1.50, *p* < 0.01, and TT vs. CC revealed OR = 2.68, *p* < 0.01 (Rmo OR = 2.05, *p* = 0.01, and Dmo OR = 1.72, *p* < 0.01); in the non-DM2 group CT vs. CC revealed OR = 1.30, *p* = 0.12, and TT vs. CC revealed OR = 1.75, *p* = 0.05 (Rmo OR = 1.38, *p* = 0.14, and Dmo OR = 1.46, *p* = 0.04). The authors concluded that the rs1801133 polymorphism was significantly associated with DR risk in the DM2 and Asian groups, especially in the Asian group (both DM types). Genetic factors seem to have more impact on Asian population.	[30]
*MTHFR*	rs1801131 (A1298C)	DM2 n = 607 DR n = 159 DNR n = 448 (AR)	A1298C *MTHFR* polymorphism increases the risk of DR developing in Arabian countries, but only in the dominant genetic model (OR = 2.96, *p* < 0.01). However, as results demonstrate a non-additive effect, an over-dominant pattern was suggested. Broader studies covering the aspect of A1298C polymorphism in DR were recommended.	[35]
*MTHFR*	rs1801133 (C677T)	DM2 n = 375 (DNR n = 196; NPDR n = 95; PDR n = 84) n = 205 CTR (H) (Pakistan)	The minor allele of rs1801133 (T) had a protective effect (OR = 0.59, *p* = 0.00228) in DM2, whereas in the case of the minor allele of rs1801131 (C) the association with DM2 was not found (OR = 1.18, *p* = 0.35). None of these SNPs showed an association with increased risk of NPDR (rs1801133 OR = 0.88, *p* = 0.59, rs1801131 OR = 1.57, *p* = 0.06) or PDR (rs1801133 OR = 0.81, *p* = 0.50, rs1801131 OR = 1.71, *p* = 0.08).	[36]
	rs1801131 (A1298C)			
*NVL*	rs142293996	Discovery stage: DM2 n = 5857; n = 3246 (Eur) DNR n = 1970, DR n = 1276 (NPDR n = 878, PDR n = 398) n = 2611 (AfA) DNR n = 941, DR n = 1670 (NPDR n = 573, PDR n = 1097) Replication stage: n = 37,708 (DM1 and DM2) n = 18,545 (Eur) DNR n = 7713, DR n = 10,832 n = 16,453 (As) DNR n = 7751, DR n = 8702 n = 2710 (His) DNR n = 1240, DR n = 1470	Seven SNPs met GWAS significance (all from PDR or extreme cases of DR; two separate loci in AfA and 5 loci in Eur). The most strongly associated variants were rs142293996 (chromosome 1) in Europeans (OR = 2.38, *p* = 2.1 × 10^−9^) and rs115523882 (chromosome 3) in African Americans (OR = 3.10, *p* = 5.4 × 10^−9^), but only rs142293996 passed meta-analysis with replication samples, after adjusting for covariates based on a Fisher exact test (extreme cases of PDR in Europeans).	[37]
*CRP*	rs2808629	DM2 n = 1018 DNR n = 400 DR n = 618 (China)	Four tagging SNPs (rs2808629, rs3093077, rs1130864 and rs2808634) within *CRP* region were genotyped for all the participants. The rs2808629 (G allele) was significantly associated with increased susceptibility to DR (OR = 1.296, *p* = 0.006). This association remained significant after adjustment for confounding factors, including HbA1c levels, duration of diabetes, systolic and diastolic blood pressure, BMI and sex (OR = 1.261, *p* = 0.030). No significant association with DR severity was observed (*p* = 0.387 for trend analysis)	[39]

EA—European American, Eur—European, AfA—African American, AsA—Asian American, HA—Hispanic American, As—Asian, NAs—non-Asian (Caucasian and African American), AR—Arabian patients from North Africa and Middle East, His—Hispanic, DNR—diabetic patients with no retinopathy, DR—diabetic retinopathy, NPDR—non-proliferative diabetic retinopathy, PDR—proliferative diabetic retinopathy, DM1—diabetes mellitus type 1, DM2—diabetes mellitus type 2, IFG—impaired fasting glucose, CTR—control group, H—healthy subjects, Rmo—recessive model, Dmo—dominant model.

### 3.2. Chromosome 2

The protein encoded by the *EHD3* gene, Eps15 homology domain-containing protein 3 (also abbreviated EHD3), is one of four EHD proteins, a family containing EH domain. The major function of EHD3 is to regulate endosomal membrane trafficking during endocytosis, but the list of the protein functions is still evolving and appears to vary depending on the tissue in which it is expressed [43]. The association of *EHD3* genetic variation (rs3754840, intronic variability, allele T) with DR has been reported by Imamura et al. [44]. EHD3 malfunction can cause heart failure, depressive disorder, glioma, or ciliopathies. As the *EHD3* expression in the retina has been reported, and the involvement of *EHD3* in the formation of cilia in the retinal pigment epithelium (RPE) cells has been demonstrated by Lu et al. [45], it could constitute a rationale for further analysis of this genetic variability association with DR [43,44,46].

The other gene—calpain-10 (*CAPN10*)—is the first diabetes gene to be identified through a genome scan, and polymorphism in its gene were indicated to be associated with type 2 diabetes as well as insulin action, insulin secretion, aspects of adipocyte biology and microvascular function [47]. However, SNP43 polymorphism in *CAPN10* tested in a Polish population was not associated with DR development [48].

Table 2 presents the most important polymorphisms related to retinopathy found on chromosome 2.

### 3.3. Chromosome 3

Peroxisome proliferator-activated receptor gamma (PPAR-γ, NR1C3) is a member of nuclear steroids receptor PPRs encoded by the *PPARG* gene. The protein is widely distributed in the human body (also in the retina) but expressed mainly in adipose tissue [49]. At least three isoforms, an effect of alternative splicing, are known. Like other PPARs, PPAR-γ forms heterodimers with retinoid X receptors (RXRs) and regulates expression of various genes, especially those engaged in fatty acids and glucose metabolism, angiogenesis, and inflammatory pathways [50]. PPAR-γ activation exerts anti-inflammatory, antioxidative, and antiproliferative effects. It could also inhibit diabetes-induced retinal leukostasis and leakage. Taking all of this together, one action of PPAR-γ is considered capable of reducing the progression of DR [51,52]. Finding that one of the missense mutations (rs1801282 C/G, known as Pro12Ala, and quite common in some populations) could influence the risk of insulin resistance and influences lipid metabolism [53] focused the attention of researchers working with DR risk factors. Studies estimating rs1801282 association with DR were performed in Europe and especially Asia. The outcome about the association of rs1801282 with DR varies. While some of them found rs1801282 protective, others found them risky, and the majority found no association. Malecki et al. [48] found that Pro12Ala polymorphism may be associated with decreased risk of DR, but the effect may be indirect. A comprehensive meta-analysis of 14 case-control studies published from 1999 to 2020 confirmed this general tendency [54] (Table 3). It should be noted, however, that authors clearly underlined the most obvious limitation of discussed studies, including, among others, study samples that are too small in size, and they often used healthy subjects-based, unstratified control groups. This leaves the question of the potential rs1801282 association with DR still open. In studies analyzing its possible association with DR pathogenesis, same as for rs1801282, no such effect (risk or protection) was found either [54] (Table 3).

Golgi integral membrane protein 4 (GOLIM4) is the product of *GOLIM4* gene, a Golgi-resident protein type II. *GOLIM4* may process proteins synthesized in the endoplasmic reticulum and assist in the transport of proteins through the Golgi apparatus. *GOLIM4* was reported to play a role in protein trafficking (endosome to Golgi), and also to have anti-apoptotic functions. Its overexpression was observed in some types of cancers, whereas depletion reduces cell proliferation and cell viability by inducing apoptosis [55]. The rs115523882 variant (allele A) located near the *GOLIM4* gene was found to be most strongly associated in African American PDR patients in the already mentioned GWAS study [37]. As with all other studied SNPs, it did not reach genome-wide significance with replication. However, it is a rare allele in people of African ancestry (MAF = 0.0393), and not common in other ethnic groups, so it could have had insufficient power to replicate it. Thus, its possible role in PDR pathogenesis cannot be excluded. According to the authors, rs115523882 variation could specifically change a motif that is a binding site for Nlx3 (a transcription factor), suggesting this SNP may play a regulatory role.

*STT3B* gene encodes a catalytic subunit of an oligosaccharyltransferase (OST) complex, which transfers oligosaccharides onto asparagine residues. Polymorphism in this gene (rs12630354) has been found significantly associated with DR in a large GWAS study in Japanese DM2 patients by Imamura et al. [44]. *STT3B* is ubiquitously expressed in human tissues, and the homozygous mutation of *STT3B*, which results in the ablation of its mRNA, and causes the congenital disorder of glycosylation (CDG1X). CDG1X is a neurological disease; the clinical manifestation of which includes multiple neurologic abnormalities, as well as a lack of visual tracking and the optic nerve hypoplasia. Since OST complex proteins, including STT3B, have been suggested to participate in local synthesis and quality control of membrane proteins involved in cholesterol and steroid metabolism in the adrenocortical cells, authors argue that rs12630354-T might affect the susceptibility to DR indirectly via the dysregulation of cholesterol and steroid metabolism in the adrenal glands. In silico analyses suggest that the risk allele, rs12630354-T, might be involved in transcriptional regulation and may contribute to DR susceptibility through a consequent upregulation of *STT3B* expression. However, as it was the first report of the novel locus associated with DR, and replication studies using Japanese, Korean, European, and African American samples failed, further analyses of this association are required [44]. Table 3 presents the most important polymorphisms related to retinopathy found on chromosome 3.

### 3.4. Chromosome 4

The 135-kD centrosomal protein (CEP135) is encoded by the *CEP135* gene, which acts as a scaffolding protein during early centriole biogenesis (serves as a platform for CEP250, and helps recruit CEP295), is required for centrioles cohesion during interphase, and maintains the structure and organization of the centrosome and microtubules. Mutations in this gene are associated with reduced neurogenesis and primary microcephaly [56]. CEP135 is an intracellular protein that is expressed also in the retina. It is considered that CEP135 can be involved in TGF-β signaling, the pathway upregulated in the aqueous humor and vitreous body during the development of proliferative diabetic retinopathy [57]. A decade ago, one of the GWAS studies evaluating genetic variation risk for severe proliferative retinopathy development in DM1 patients (Americans of European descent) found a strong association at rs4865047 within the intronic region of the *CEP135* gene, although with no genome-wide significance [7]. The meaning of C/T allele variation is not known yet. Although authors found also that rs4865047 is in a high linkage disequilibrium (LD) with a number of SNPs in intronic regions within the *CEP135* gene, but not with exonic SNPs or loci that are related to the variation in expression of mRNAs (eQTLs). Rs4865047 risk allele T association with rapidly progressive PDR has been confirmed in East European DM1 patients [57] (Table 4).

Encoded by the *NPY2R* gene neuropeptide Y receptor type 2 (NPY2R) is a G-protein coupled receptor, one of five for neuropeptide Y (NPY). Both NPY and NPY2R play important roles in diabetic retinopathy and retinal neovascularization [58]. The rs1902491 (A/G) localized in the intergenic region, 75 kb upstream of *NPY2R* gene, has been found as the most associated with PDR in DM1 subjects in one of the American GWAS study [7]. However, despite maintaining the tendency of the protective effect for minor allele G significant association for rapid progressive PDR was not obtained in the Lithuanian study [57] (Table 4).

### 3.5. Chromosome 5

The search results failed to identify genes associated with retinopathy on chromosome 5.

### 3.6. Chromosome 6

Chromosome 6 involves one important gene (*VEGF*) that is suggested as risk factors for diabetes retinopathy. Vascular endothelial growth factor (VEGF) is considered one of the determinants of DR development. It is a mitogen of endothelial cells. Hyperglycemia occurring in diabetes induces increased expression of *VEGF*. High concentration of VEGF in the vitreous causes increased retinal vascular permeability and neovascularization, which directly contributes to the development of DR [59]. Under the general name VEGF, there are as many as seven isoforms of this protein. Anti-VEGF intravitreal therapies combine anti-angiogenic and anti-edematous activities. Although the use of anti-VEGF in the treatment of DR is a huge step forward, there is extensive discussion in the literature about the role of anti-VEGF as a substitute for panretinal photocoagulation. Based on current data, laser therapy can be avoided if a perfectly planned anti-VEGF therapeutic strategy can be used [60]. Two SNPs showed discordant results in different studies. For rs2010963, Awata et al. [61] found this polymorphism to be a risk factor for developing DR. Different results were obtained by Uthra et al. [62] and Yang et al. [63]. Similar results were obtained for rs 302539, whereas Kim et al. [64] showed a high correlation of polymorphism with DR development and, in two other studies, no effect of polymorphism with DR was found [62,64]. Szaflik et al. [65] (2008) analyzed two polymorphisms (−634 G/C, rs2010963; −460 C/T) in the promoter region. According to these data, polymorphism −634C may be associated with promoter activity and serve as a predictive factor for DR development. On the other hand, Buraczyńska et al. [66] found no correlation between snp (+405) in *VEGF* gene and diabetic complications in type 2 diabetes patients.

The most important data are present in Table 5.

### 3.7. Chromosome 7

Chromosome 7 involves four important genes (*IL-6*, *eNOS*, aldolase reductase, and *PAI-1*) that are suggested as risk factors for diabetes retinopathy.

Multiple studies have found DR to have an immunologic basis. High levels of pro-inflammatory interleukin-6 (IL-6) have been observed in the vitreous body of patients with longstanding diabetes with DR [67]. Lu et al. [45] analyzed two SNP in the *IL-6* gene. This study found that the polymorphism in rs1800795 and rs1800796 might point to a relatively high risk for DM2 patients suffering from PDR in a Chinese population.

Endothelial nitric oxide synthase (eNOS) is synthesized by the cells of the vascular endothelium and is directly related to maintaining the tone of the blood vessels. Inactivation of *eNOS* gene in mice caused hypertension [68], and the role of NO is suggested in the regulation of basal or stimulated vasodilation [69]. In the gene encoding eNOS, the association of two SNPs with the development of DR was analyzed. Bazzaz et al. [70] found a significant relationship between the polymorphism in rs2070745 and the occurrence of diabetic retinopathy in patients with type 1 diabetes. In another study, Momeni et al. [71] analyzed the correlation between polymorphism G894T in *eNOS* gen with DR in patients with diabetes. This study showed that this polymorphism was more common in people with type 2 diabetes, but no correlation was found between this polymorphism and retinopathy and proteinuria.

The gene located on chromosome 7 encodes aldolace reductase (AR). AR is an enzyme of the polyol pathway where glucose is converted to sorbitol [72]. In addition, AR induces vascular spasms and hemodynamic pathogenic changes that cause DR. The accumulation of sorbitol in the cells of the retina leads to osmotic damage to the retinal blood vessels to directly cause the development of DR [73]. Some AR inhibitors as drug candidates have been developed for treatment of diabetic complications [74]. In a study on an Egyptian population the role of C106T rs759853 polymorphism was excluded in conferring the risk of DR. As indicated by the authors of this study, this polymorphism was not an independent genetic risk factor for DR [75].

The *PAI-1* gene located on chromosome 7 encodes the plasminogen activator inhibitor-1. It is known that high activity of this gene is associated with atherosclerosis and thromboembolism [76]. In addition, a higher concentration of PAI-1 was observed in the serum of diabetics. This protein is involved in the microvascular occlusion of the retina and thus contributes to the development of DR in diabetics [77]. The correlation between retinopathy and type 2 diabetes was also analyzed by Khan et al. [78]. Authors have conducted a molecular analysis of the association of both the *ACE* and *PAI-1* genes with diabetic retinopathy and diabetic patients without retinopathy (DNR) in 592 samples consisting of: 196 patients with DR, 200 DNR and 196 controls, respectively. It was mentioned that the polymorphism (rs4646994) was responsible for approximately 50% of the inter-individual variability in plasma ACE levels (Section 3.17 and table within), and individuals with the D/D genotype had significantly higher levels compared to those with the I/I or I/D genotypes. As a result of the analysis, Khan et al. [78] have suggested that changes in both *ACE* and *PAI-1* gene polymorphisms in DR, DNR and DM2 patients are risk factors that may be useful as prognostic markers. On the other hand, the same study showed no significant association of the *PAI-1* gene rs1799768 with the development and progression of DR in patients with type 2 diabetes. The most important data are present in Table 6.

### 3.8. Chromosome 8

The search results failed to identify genes associated with diabetic retinopathy on chromosome 8.

### 3.9. Chromosome 9

Intronic rs14050842 in *PALM2* (paralemmin 2, a member of a multigene family consisting of two other members, paralemmin (*PALM*) and palmdelphin (*PALMD*)) gene located on chromosome 9 was typed as predisposed to DR. The results are shown in Table 7.

### 3.10. Chromosome 10

Using statistical models, Huang et al. [79] identified six SNPs located on chromosome 10 associated with DR. A genome-wide association study was performed involving 749 unrelated DM2 subjects (174 with DR and 575 without DR) and 100 non-diabetic controls of the Han Chinese population residing in Taiwan. One SNP was located on *PLXDC2* (plexin domain-containing 2) gene, which may play a role in tumor angiogenesis, fasting insulin levels and insulin resistance. Three SNPs were found on *ARHGAP22* (Rho GTPase-activating protein 22) gene, which was shown to be involved in the signal transduction pathway that regulates endothelial cell capillary tube formation during angiogenesis with a role in tumor cell movement and may be involved in a novel insulin-regulated pathway.

Renalase (RNLS) is an enzyme with monoamine oxidase activity that metabolizes circulating catecholamines and is involved in the regulation of blood pressure [80]. According to Buraczyńska et al. [81], Asp37Glu missense polymorphism (rs2296545) in the *RNLS* gene affects susceptibility to diabetic retinopathy in type 2 diabetes mellitus.

The results are shown in Table 8.

### 3.11. Chromosome 11

The search results failed to identify genes associated with diabetic retinopathy on chromosome 11.

### 3.12. Chromosome 12

The search results failed to identify genes associated with diabetic retinopathy on chromosome 12.

### 3.13. Chromosome 13

An analysis of the genetic explanation for diabetic retinopathy was undertaken by Huang et al. [79]. The authors studied the genes that increase the risk of DR in type 2 diabetes and attempted to elucidate the mechanism underlying the pathogenesis of retinopathy. A genome-wide association study was performed involving 749 unrelated DM2 subjects (174 with DR and 575 without DR) and 100 non-diabetic controls. A total of 12 SNPs were statistically analyzed, including *HS6ST3* (heparan sulfate 6-O-sulphotransferase 3) gene located on chromosome 13q. Heparan sulfate (HS) sulfotransferases are proteins that influence the formation of structures for interaction with various proteins, which then modulate cell proliferation and differentiation, adhesion, migration, inflammation, blood coagulation and other various processes [82]. The authors found that the tested gene *HS6ST3* (OR, 2.33; 95% CI, 1.13–4.77), but also *MYSM1* (Myb-like, SWIRM, and MPN domains 1) located on chromosome 1p (OR, 1.50; 95% CI, 1.03–2.20), and both *PLXDC2* (plexin domain-containing 2; OR, 1.67; 95% CI, 1.06–2.65), and *ARHGAP22* (Rho GTPase-activating protein 22; OR, 1.65; 95% CI, 1.05–2.60) located on chromosome 10q are involved in the pathogenesis of DR, which may be a factor to monitor this process (Table 9).

### 3.14. Chromosome 14

No genes associated with retinopathy on chromosome 14 were identified in the search results.

### 3.15. Chromosome 15

*MYO5C* (Myosin VC) gene polymorphism (rs3751624) was shown to be associated with diabetes retinopathy by Hsieh et al. [83]. Results are shown in Table 10.

### 3.16. Chromosome 16

GWAS study results presented by Grassi et al. [7] identified novel genetic loci associated with the sight threatening complications of diabetic retinopathy. Among 2,543,887 single nucleotide polymorphisms (SNPs) tested, several SNPs on chromosome 16 were found in genes *CCDC101, NUPR1, SULT1A1, SULT1A2, ZNRF1* with top association (meta OR = 0.68) for *A2BP1* (Ataxin 2-Binding Protein 1) gene, and RNA-binding protein involved in regulating tissue-specific alternative splicing [84] (Table 11).

### 3.17. Chromosome 17

A 2014 study by Wang et al. [11] discovered a new role for the *FAM18B* gene in diabetic retinopathy. The gene is located on chromosome 17 in cytogenetic band 17p11.2 [85]. In search of genetic elements underlying diabetic retinopathy, a genome-wide association study was performed [7,86]. An interesting finding of the analysis was the association of diabetic retinopathy with a SNP, rs11871508, in the coding Family Sequence Similarity 18, Member B (*FAM18B*), also known as trans-Golgi Network Vesicle Protein 23 Homolog B (TVP23B). The function of *FAM18B* is unknown. However, the encoded protein is thought to be an integral membrane protein. Since it is associated with diabetic retinopathy, it was needed to determine the possible role of FAM18B in diabetic retinopathy. The role of FAM18B was regulating human retinal microvascular endothelial cell viability, migration, and endothelial tube formation was determined after RNAi-mediated knockdown of *FAM18B*. The presence of *FAM18B* was determined by qRT-PCR in CD34+/VEGFR2+ mononuclear cells isolated from a cohort of 17 diabetic patients with and without diabetic retinopathy [11]. Overall, these findings support a role for *FAM18B* in the pathogenesis of diabetic retinopathy.

One of the most interesting candidate genes related to diabetic retinopathy (DR) is angiotensin-converting enzyme (*ACE*) gene, which has been subjected to many analyses.

An insertion/deletion (I/D) polymorphism correlation analysis in the *ACE* gene with DR in a Chinese population was performed. The results described earlier were inconclusive; therefore, a meta-analysis was performed, taking into account 17 studies with a total of 1039 cases and 1185 controls [87]. Although the mechanisms underlying DR are not fully understood, it is known that factors of the renin-angiotensin system (RAS) are involved in the process. Previous studies by other authors have shown that patients with diabetes and retinopathy have elevated serum levels of ACE, prorenin and renin [88]. This inspired the analysis of polymorphisms in the genes of these factors to determine the genetic determinants of the occurrence of retinopathy. The association of the *ACE* I/D polymorphism with the DR was estimated by calculating the cumulative OR and 95% CI in the codominant, dominant and recessive genetic model, respectively. Finally, Lu et al. [87] determined that the I/D polymorphism of the D allele may increase the risk of DR, although the biological mechanism remains unclear. However, the authors emphasize that serum ACE concentration is significantly higher in carriers of the D/D genotype than in carriers of the I/D or I/I genotype, and, in addition, the final component of RAS has a vasoconstrictor effect, promotes the accumulation of extracellular matrix and induces the production of PAI-1 in endothelial cells [89].

Similar analyzes were carried out by Nikzamir et al. (2010), who have investigated the relationship between DR and angiotensin converting enzyme insertion/deletion gene polymorphism in Iranian patients with type 2 diabetes without overt nephropathy. The authors analyzed not only the *ACE* gene polymorphism, but also the following variables: age, gender, body mass index, duration of diabetes, medications used, history of coronary artery disease and its complications, blood pressure, glycemia, hemoglobin A1c, total cholesterol, HDL, LDL, triglycerides, plasma creatinine, and 24-h urinary albumin excretion. Type 2 diabetic patients with DR (n = 178) and type 2 diabetic patients without DR (n = 206) were studied, and these two groups differed in diabetes duration (*p* = 0.037), ACE activity (*p* < 0.001) and *ACE* genotype (*p* = 0.008). Finally, the authors suggested that the DD genotype was significantly more common in patients with retinopathy (*p* = 0.009) [90].

Other researchers have analyzed *ACE* gene polymorphism in non-proliferative (NPDR, n = 136) and proliferative retinopathy (PDR, n = 94). As a result, the DD genotype of the angiotensin-converting enzyme polymorphism was shown to be more common in the PDR group (*p* < 0.001). Results suggest that the DD genotype increases ACE activity, which in turn increases the risk of proliferation in diabetic retinopathy. The authors concluded that the DD genotype, but not the ID genotype, was associated with an increased chance of PDR, suggesting a recessive mode of inheritance in the D allele of the *ACE* gene polymorphism [91].

The importance of gene polymorphism in the development of retinopathy was also investigated by Liang et al. (2013) [92], who investigated the relationship of the 2350 G/A polymorphisms of *ACE*. The authors emphasize that the renin-angiotensin system is involved in the development of DR, and RAS inhibition is important in delaying the progression of the disease. A total of 145 DM2 patients took part in the study and the results were compared with the control group (n = 90). Finally, Liang et al. [92] concluded that *ACE* 2350 G/A polymorphism is related to DR in Han Chinese patients with type 2 diabetes, but this relationship should be individually analyzed in other ethnic groups.

Also, rs9896052 in *GRB2* gene that binds phosphorylated insulin receptor substrate 1 was found to have an association with diabetic retinopathy [93].

Table 12 presents the most important polymorphisms related to retinopathy found on chromosome 17.

### 3.18. Chromosome 18

The search results failed to identify genes associated with diabetic retinopathy on chromosome 18.

### 3.19. Chromosome 19

Chromosome 19 involves two of the most important genes (*APOE* and *ICAM-1*) that are suggested as risk factors for diabetes retinopathy. Apolipoprotein-E (*ApoE*)—mapped on human chromosome 19 with three major alleles (E2, E3, E4) and six possible genotypes (E2/2, E2/3, E2/4, E3/3, E3/4, E4/4)—is a candidate gene for the development of Type 2 diabetes mellitus [94], plays important role in lipid formation and is associated with plasma lipid and lipoprotein levels [95]. Dlouha et al. [96] showed that *APOE4* allele revealed an association with retinopathy, and carriers of at least one gene allele are protected against DM2 related retinopathy (OR [95% CI] = 0.65 [0.42–0.99]. On the other hand, Liew et al. [12], Errera et al. [97] and Shcherbak [98] showed that there is no association between *APOE* gene polymorphisms e2/e3/e4 with diabetic retinopathy (Table 11).

Intercellular adhesion molecule-1 (*ICAM-1*)—a member of the adhesion immunoglobulin super family that is well-known—was identified to have a critical molecule secreted by endothelium during vascular inflammation and is responsible for formation, growth and rupture of atheroma [99]. Also, in the aspect of leucocyte adhesion to the diabetic retinal vasculature it has been implicated in the pathogenesis of diabetic retinopathy [100]. *ICAM-1* 469 (K/K, K/E, E/E) genotype KK was shown to be a genetic risk factor for retinopathy in Type 2 diabetes mellitus [101]. Table 13 presents the most important polymorphisms related to retinopathy found on chromosome 19.

### 3.20. Chromosome 20

According to Huang et al. [102] seven SNPs were found on chromosome 20 which may be involved in diabetic retinopathy epidemiology, but only rs761207 and rs6031415 in junctophilin 2 gene (*JPH2*) were found to have increased the risk for disease development.

Protein tyrosine phosphatase non-receptor type 1 (*PTPN1*) encodes the protein tyrosine phosphatase 1 B that is an inhibitor of insulin signaling acting, plays a critical role in regulating the responses of eukaryotic cells to multiple extracellular signals, such as hormones, cytokines, and growth factors, and is shown to be associated with diabetes mellitus disease [103]. However, in a Polish population, Malecki [48] found no association between two SNPs (rs3787345, rs754118) present in *PTPN1* gene and diabetic retinopathy development.

The results are shown in Table 14.

### 3.21. Chromosome 21

The search results failed to identify genes associated with diabetic retinopathy on chromosome 21.

### 3.22. Chromosome 22

Tissue inhibitor of metalloproteinase-3 (*TIMP-3*) gene is a member of the tissue inhibitor of metalloproteinases (TIMPs) family composed of four members (TIMP1, TIMP2 TIMP3, TIMP4), physiological inhibitors of matrix metalloproteinases (MMPs) [104] with a significant role in the control of extracellular matrix remodeling. TIMP-3 is unique among the four TIMPs due to its extracellular matrix (ECM)-binding property and might play a role in the regulation of retinal neovascularization during the progression of diabetic retinopathy, but polymorphisms in the promoter region of the gene were not associated with proliferative diabetic retinopathy [105] (Table 15).

### 3.23. Chromosome X/Chromosome Y

The search results failed to identify genes associated with diabetic retinopathy on chromosome X/Y.

## 4. Conclusions

The etiopathology of diabetic retinopathy is influenced by the disturbance of biochemical metabolic pathways and genetic factors, including gene polymorphism. The presented information shows that the genes associated with the pathogenesis of diabetic retinopathy are located on several chromosomes, and their polymorphic variants (SNPs) are associated with the development of the disease. However, data on several chromosomes are still unclear and require more research. According to the literature, the most important genes associated with diabetic retinopathy are located on chromosomes 1–22, except for chromosomes 5, 8, 11, 12, 14, 18, 21, X/Y. The largest number of genes related to the development of retinopathy is found on chromosome 1 (*SELP*, *MTHFR*, *NVL*, *CRP*), and 7 (*IL-6*, *eNOS*, *AR*, *PAI-1*), while most studies on the relationship between the development of retinopathy and polymorphism are focused on *VEGF* gene (chromosome 6), *ACE* (chromosome 17), and *APOE* (chromosome 19). The publications presented in this review indicate polymorphisms determined by various genetic methods, including GWAS, a method that searches for correlations among many thousands of SNPs. The search for candidate genes for disease markers is associated with a future approach involving faster diagnosis, but also disease prevention. Patients at risk of developing diabetic retinopathy will be able to follow the rules that will decrease the development of the disease and will allow monitoring of the process.

This article indicates both the role of specific genes in the development of diabetic retinopathy and polymorphic changes within the indicated genes that may have an impact on exacerbating the symptoms of the disease. The goal of our work was also to collect and systematize information on the role of SNPs in DR. SNPs can affect the encoded protein. If they are present in the coding sequence and lead to an amino acid change (referred to as a non-synonymous SNP or mutation), they may modify the protein’s activity or have the potential to alter all steps of gene expression depending on their genomic location. If present in transcriptional regulatory elements, they can influence mRNA expression. SNPs created in genes can affect mRNA splicing, nucleocytoplasmic export, stability and translation. We organized the data according to chromosome number. Several of the genes discussed above have been suggested as likely to function together in linkages due to their close location on the chromosome, but most have not demonstrated such linkages.

The list of polymorphisms may allow for a broader genotyping of the population in order to clarify the obtained results for other researchers. We believe that this publication will systematize knowledge on the topic and allow scientists to plan research leading to wide potential outcomes.

## Figures and Tables

**Table 2:** Polymorphisms of genes related to diabetic retinopathy on chromosome 2.

Gene	Polymorphism or rs ID Number	No. of Participants	Results	References
*EHD3*	rs3754840	DM2 n = 14,080 in total; Discovery stage: DR set-1 n = 5415, set-2 n = 790, DNR set-1 n = 4676, set-2 n = 1779 Validation stage: DR n = 2260, DNR n = 723 Replication stage: DR n = 750, DNR n = 839 (Japan)	Results revealed *EHD3* as a novel susceptibility gene to DR (allele T) (association *p* = 2.17 × 10^−6^ < 2.71 × 10^−6^ = 0.05/18480).	[44]
*CAPN10*	SNP43	DNR n = 238 DR n = 121	Distribution of genotypes: DR (46.3% GG; 41.3% GA; 12.4% AA); DNR (45.8% GG; 45.0% GA; 9.2% AA); *p* = 0.60	[48]

DNR—diabetic patients with no retinopathy, DR—diabetic retinopathy, DM2—diabetes mellitus type 2.

**Table 3:** Polymorphisms of genes related to diabetic retinopathy on chromosome 3.

Gene	Polymorphism or rs Number	No. of Participants	Results	References
*PPAR γ*	rs1801282 (Pro12Ala)	DR n = 4369 CTR n = 5658 (DNR + H)	The two common SNPs were analyzed: rs1801282 C/G, and rs3856806 C/T. The pooled results did not reveal an association between PPAR-γ rs1801282 C/G and DR susceptibility in the overall population. Not significant was also race-based stratification (Caucasian and Asian), the HWE status, control design, or DR subtype stratification. Same as above, the pooled results indicated no association between the rs3856806 C/T polymorphism and DR susceptibility in the overall population in any of the five genetic models (OR = 0.74, and *p* = 0.19).	[54]
	rs3856806	DR n = 902 CTR n = 845 (DNR + H)		
*PPAR γ*	rs1801282 (Pro12Ala)	DNR n = 238 DR n = 121	Distribution of genotypes: DR (71.1% Pro/Pro; 25.6% Pro/Ala; 3.3% Ala/Ala); DNR (66.4% Pro/Pro; 30.7% Pro/Ala; 2.9% Ala/Ala). Pro12Ala might be associated with decreased risk of DR. Effect may be indirect.	[48]
*SST3B*	rs12630354	DM2 in total n = 14,080; Discovery stage: DR set-1 n = 5415, set-2 n = 790, DNR set-1 n = 4676, set-2 n = 1779 Validation stage: DR n = 2260, DNR n = 723 Replication stage: DR n = 750, DNR n = 839 (Japan)	The authors identified new SNP loci 73 kbp upstream of STT3B predisposing to DR (OR = 1.17, *p* = 1.62 × 10^−9^). Risk allele T might be involved in transcriptional regulation and may contribute to DR susceptibility through a consequent upregulation of STT3B expression.	[44]
*GOLIM4* (nearest gene)	rs115523882	Discovery stage: DM2 n = 5857; n = 3246 (Eur) DNR n = 1970, DR n = 1276 (NPDR n = 878, PDR n = 398) n = 2611 (AfA) DNR n = 941, DR n = 1670 (NPDR n = 573, PDR n = 1097) Replication stage: n = 37,708 (DM1 and DM2) n = 18,545 (Eur) DNR n = 7713, DR n = 10,832 n = 16,453 (As) DNR n = 7751, DR n = 8702 n = 2710 (His) DNR n = 1240, DR n = 1470	The rs115523882 was the most strongly associated variants in African American PDR patients (OR = 3.10, *p* = 5.4 × 10^−9^), but with no genome-wide significance in replication (possibly insufficient power to replicate caused by a low frequency in other ethnic groups).	[37]

Eur—European, AfA—African American, As—Asian, His—Hispanic, DNR—diabetic patients with no retinopathy, DR—diabetic retinopathy, NPDR—non-proliferative diabetic retinopathy, DM1—diabetes mellitus type 1, DM2—diabetes mellitus type 2, PDR—proliferative diabetic retinopathy, CTR—control group, H—healthy subjects.

**Table 4:** Polymorphisms of genes related to diabetic retinopathy on chromosome 4.

Gene	Polymorphism or rs Number	No. of Participants	Results	References
*CEP135*	rs4865047	DM1 n = 469 PDR n = 208 NPDR n = 261 (EA)	No associations were significant at a genome-wide level, but the top association was at rs4865047 (OR = 0.65, *p* = 0.11, P_CA_ = 2.06 × 10^−5^).	[7]
*CEP135*	rs4865047	DM1 n = 132 PDR n = 48 NPDR (CTR) n = 84 H n = 90	The distribution of rs4865047 (*CEP135*) genotypes was significantly different between PDR and NPDR when compared with the reference group (*p* < 0.001), with no detection of minor allele homozygotes (TT) in either group. MAF in PDR and NPDR were much higher than in the reference group (OR = 4.325, *p* = 0.001 and OR = 6.089, *p* < 0.0001 respectively). The risk prediction in the codominant model showed that CT carriers of minor alleles had a seven-fold higher odds ratio of PDR when compared with wild-type homozygous carriers CC and when compared to the reference group (OR = 7.2, *p* = 0.001).	[57]
*NPY2R*	rs1902491	DM1 n = 469 PDR n = 208 NPDR n = 261 (EA)	The analysis showed that the top association was at rs1902491 (OR = 0.81, *p* = 0.13, P_CA_ = 2.81 × 10^−5^), a new locus associated with PDR.	[7]
*NPY2R*	rs1902491	DM1 n = 132 PDR n = 48 NPDR (CTR) n = 84 H n = 90	There were no significant differences in the distribution of rs1902491 genotype frequencies (PDR and NPDR versus the reference group, *p* > 0.05). MAF of rs1902491 did not differ significantly between the groups (*p* > 0.05). The risk prediction in additive, recessive and codominant models showed the possible protective effect against PDR, but without significance.	[57]

EA—European American, NPDR—non-proliferative diabetic retinopathy, PDR—proliferative diabetic retinopathy, DM1—diabetes mellitus type 1, CTR—control group, H—healthy subjects, P_CA_—Combined Analysis *p* value.

**Table 5:** Polymorphisms of genes related to retinopathy present on chromosome 6.

Gene	Polymorphism or rs Number	No. of Participants	Results	References
*VEGF*	+405	DM2 n = 426 (DR n = 195) CTR n = 493	+405 genotype was not associated with diabetic complications in type 2 diabetes patients.	[66]
	rs2010963 −634 G/C	DNR n = 215 (PDR n = 82; NPDR n = 72) CTR n = 61	The G/C polymorphism genotype distribution and the frequency of the C allele were significantly higher in the NPDR group than in control patients (OR = 1.69, 95% CI = 1.03–2.79). Analysis of the distribution of combined genotypes of the *VEGF* gene revealed the prevalence of the C/C-C/C genotype in NPDR patients (OR = 8.26, 95% CI = 1.79–37.99) and C/G-CC in PDR patients (OR = 3.36, 95% CI = 1.39–8.12).	[65]
	−460 C/T rs833061		No association between the C/T polymorphism and diabetic retinopathy.	
	rs2010963	DNR (CTR) n = 118 DR n = 150	Among seven common polymorphisms in the promoter region, 5-untranslated region (UTR) and 3UTR of the *VEGF* gene, genotype distribution of the C(634)G polymorphism differed significantly (*p* = 0.011) between DR and CTR, and the C allele was significantly increased in patients with retinopathy compared with those without retinopathy (*p* = 0.0037). The odds ratio (OR) for the CC genotype of C(634)G to the GG genotype was 3.20 (95% CI 1.45–7.05, *p* = 0.0046).	[61]
	rs201963	n = 213 (DNR n = 87; DR n = 44 and n = 82 with no DR evidence)	No significant association was observed between genotypes, alleles and haplotypes of −634C/G polymorphisms and DR or its severity.	[62]
	rs2010963	DM2 total n = 268 (DNR n = 139; DR n = 129)	No significant association was observed between analyzed group (*p* = 0.67)	[63]
	rs3025039	n = 213 (DNR n = 87; DR n = 44 and n = 82 with no DR evidence)	No significant association was observed between genotypes, alleles and haplotypes of 936C/T polymorphisms and DR or its severity.	[62]
	rs3025039	DR n = 41 DNR n = 118	A higher frequency of the TT genotype was observed in patients with proliferative diabetic retinopathy (*p* = 0.02). VEGF 936 C/T polymorphism was associated with plasma levels of VEGF, and DM2 patients with DR are characterized by a particular VEGF genotype and have higher VEGF levels than DNR and the general healthy subjects.	[64]
	rs3025039	DM2 total n = 268 (DNR n = 139; DR n = 129)	No significant association was observed between analyzed group (*p* = 0.93)	[63]
	rs833069		No significant association was observed between analyzed group (*p* = 0.74)	
	rs699949		It was found to have a significant association with DR (OR = 3.54 CI = 1.12–11.19)	
	rs833061		It was found to have a significant association with DR (OR = 3.72 CI = 1.17–11.85)	
	rs13207351		It was found to have a significant association with DR (OR = 3.76 CI = 1.21–11.71)	
	rs2146323		It was found to have a significant association with DR (OR = 2.25 CI = 0.81–6.29)	
	rs3025021		It was found to have no association with DR (OR = 1.08 CI = 0.26–4.44)	

DNR—diabetic patients with no retinopathy, DR—diabetic retinopathy, DM2—diabetes mellitus type 2, CTR—control group, NPDR—non-proliferative diabetic retinopathy, PDR—proliferative diabetic retinopathy.

**Table 6:** Polymorphisms of genes related to retinopathy present on chromosome 7.

Gene	Polymorphism or rs Number	No. of Participants	Results	References
*IL-6*	rs1800795	DR (DM2) n = 215 DNR n = 207	DR patients with PDR had a significantly higher frequency of IL-6 –174 GC (OR 0.58; 95% CI 0.34–0.99; *p* = 0.011) than DNR.	[67]
	rs1800796		DR patients with PDR had a significantly higher frequency of IL-6 –572 GG (OR 0.53; 95% CI 0.24–1.14; *p* = 0.016) than DNR.	
*eNOS*	rs2070745	DR (DM1) n = 249 CTR n = 100	The significant difference was observed between diabetic patients and healthy controls [CC + CT vs. TT *p* = 0.05, OR = 1.5, 95% CI 0.9–2.5]. The genotype frequencies for eNOS gene polymorphism were also significantly different between diabetic retinopaths and healthy controls [CC + CT vs. TT *p* = 0.0000 OR = 3.4, 95% CI 1.9–6.1].	[70]
	G894T	DR n = 94 CTR n = 94	There was no difference between prevalence of TT, GT or GG genotype based on age and sex. There was no correlation between DR or proteinuria and genotypes of eNOs.	[71]
*AR*	rs759853 (C106T)	DNR n = 120 DR n = 160	There was no correlation between rs759853 polymorphism and DR in Egyptian population.	[75]
*PAI-1*	rs1799768	DR n = 196 DNR n = 200 CTR n = 196	Genetic analysis for *PAI-1* gene polymorphism indicates that the frequency of *PAI-1* rs1799768 genotypes in DR and DNR compared to controls is not significant.	[78]

DNR—diabetic patients with no retinopathy, DR—diabetic retinopathy, PDR—proliferative diabetic retinopathy, DM1—diabetes mellitus type 1, DM2—diabetes mellitus type 2, CTR—control group.

**Table 7:** Polymorphisms of genes related to diabetic retinopathy on chromosome 9.

Gene	Polymorphism or rs Number	No. of Participants	Results	References
*PALM2*	rs14050842	DM2 in total n = 14,080; Discovery stage: DR set-1 n = 5415, set-2 n = 790, DNR set-1 n = 4676, set-2 n = 1779 Validation stage: DR n = 2260, DNR n = 723 Replication stage: DR n = 750, DNR n = 839	New SNP on *PALM* gene was identified as predisposing to DR (*p* = 4.19 × 10^−8^, OR = 1.61, 95% CI 1.36–1.)	[36]

DNR—diabetic patients with no retinopathy, DR—diabetic retinopathy, DM2—diabetes mellitus type 2.

**Table 8:** Polymorphisms of genes related to retinopathy present on chromosome 10.

Gene	Polymorphism rs Number	No. of Participants	Results	References
*RNLS*	rs2296545	DM2 n = 860 DNR n = 405; DR n = 328 CTR n = 400	Retinopathy subgroup showed a significantly higher frequency of G allele (OR 1.4, 95% CI 1.16–1.72, *p* = 0.0005) and GG genotype (OR 1.86, 95% CI 1.26–2.75, *p* = 0.001). SNP might be considered a risk factor for diabetic retinopathy in DM2 patients	[81]
*PLXDC2*	rs1571942 (C/T)	DM2 n = 749, DR n = 174, DNR n = 575, CTR n = 100	Risk allele C. A 1.67-fold greater risk of developing of DR (OR, 1.67; 95% CI, 1.06–2.65)	[79]
*ARHGAP22*	rs4838605 (C/T)		Risk allele C. A 1.58-fold risk greater risk of developing of DR (95% CI, 1.00–2.52)	
*ARHGAP22*	rs11101355 (T/C)		Risk allele T. A 1.65-fold greater risk of developing of DR (95% CI, 1.05–2.60).	
*ARHGAP22*	rs11101357 (A/G)		Risk allele A. A 1.65-fold greater risk of developing of DR (95% CI, 1.05–2.60).	
Unknown nearest gene	rs12219125 SNP (T/G)		Risk allele T. A 1.62-fold increase in DR risk (95% CI, 1.02–2.58).	
Unknown nearest gene	rs4462262 SNP (C/T)		Risk allele C. A 1.54-fold increase in DR risk (95% CI, 0.79–2.99).	

DNR—diabetic patients with no retinopathy, DR—diabetic retinopathy, DM2—diabetes mellitus type 2, CTR—control group.

**Table 9:** Polymorphisms of genes related to retinopathy present on chromosome 13.

Gene	Polymorphism rs Number	No. of Participants	Results	References
*HS6ST3* (and *MYSM1*, *PLXDC2*, *ARHGAP22*)	rs2038823	DM2 n = 749, DR n = 174, DNR n = 575, CTR n = 100	Significant associations were identified in chromosome regions 1, 5, 10 and 13 after adjusting for diabetes duration and HbA1C levels. The results suggest that *HS6ST3*, and also *MYSM1*, *PLXDC2*, *ARHGAP22*, and an unknown gene on chromosome 5q are involved in the pathogenesis of diabetic retinopathy.	[79]
*PLXDC2*	rs1571942 (C/T)		Risk allele C. A 1.67-fold greater risk of developing of DR (OR, 1.67; 95% CI, 1.06–2.65)	
*ARHGAP22*	rs4838605 (C/T)		Risk allele C. A 1.58-fold risk greater risk of developing of DR (95% CI, 1.00–2.52)	
*ARHGAP22*	rs11101355 (T/C)		Risk allele T. A 1.65-fold greater risk of developing of DR (95% CI, 1.05–2.60).	
*ARHGAP22*	rs11101357 (A/G)		Risk allele A. A 1.65-fold greater risk of developing of DR (95% CI, 1.05–2.60).	
Unknown nearest gene	rs12219125 SNP (T/G)		Risk allele T. A 1.62-fold increase in DR risk (95% CI, 1.02–2.58).	
Unknown nearest gene	rs4462262 SNP (C/T)		Risk allele C. A 1.54-fold increase in DR risk (95% CI, 0.79–2.99).	

DNR—diabetic patients with no retinopathy, DR—diabetic retinopathy, DM2—diabetes mellitus type 2, CTR—control group.

**Table 10:** Polymorphisms of genes related to retinopathy present on chromosome 15.

Gene	Polymorphism or rs Number	No. of Participants	Results	References
*MYO5C*	rs3751624	NPDR n = 102 PDR n = 72 DNR (DM2) n = 573	Allele A of rs3751624 SNP is associated with higher susceptibilities to DR	[83]

DNR—diabetic patients with no retinopathy, NPDR—non-proliferative diabetic retinopathy, PDR—proliferative diabetic retinopathy.

**Table 11:** Polymorphisms of genes related to retinopathy present on chromosome 16.

Gene	Polymorphism or rs Number	No. of Participants	Results	References
*A2BP1*	rs7355553 (A/G)	DR (DM1) n = 973 CTR n = 1856	Meta OR = 0.68, *p*-value 6.4 × 10^−7^	[7]
*CCDC101*	rs151320 (A/G)		Meta OR = 0.575, *p*-value 3.1 × 10^−6^	
*NUPR1*	rs151227 (C/T)		Meta OR = 0.575, *p*-value 3.2 × 10^−6^	
*CCDC101*	rs151229 (C/T)		Meta OR = 0.575, *p*-value 3.2 × 10^−6^	
*CCDC101*	rs151230 (C/T)		Meta OR = 0.575, *p*-value 3.2 × 10^−6^	
*CCDC101*	rs11641853 (C/T)		Meta OR = 0.576, *p*-value 3.4 × 10^−6^	
*CCDC101*	rs10521145 (C/T)		Meta OR = 0.575, *p*-value 3.4 × 10^−6^	
*ZNRF1*	rs17684886 (A/T)		Meta OR = 0.559, *p*-value 6.8 × 10^−6^	
*SULT1A1*	rs11074904 (C/T)		Meta OR = 0.557, *p*-value 7.8 × 10^−6^	
*SULT1A2*	rs11647881 (G/T)		Meta OR = 0.585, *p*-value 8.6 × 10^−6^	

DR—diabetic retinopathy, DM1—diabetes mellitus type 1, CTR—control group.

**Table 12:** Polymorphisms of genes related to retinopathy present on chromosome 17.

Gene	Polymorphism or rs Number	No. of Participants	Results	References
*FAM18B* (gene ID 51030); (*TVP23B*)	rs11871508 (G>A)	PDR n = 8 DNR n = 9	Factors such as hyperglycemia, VEGF (vascular endothelial growth factor) and AGE (advanced glycation end products) downregulated *FAM18B* expression in primary human retinal microvascular endothelial cells. CD34+ /VEGFR2+ mononuclear cells from patients with PDR showed significantly reduced *FAM18B* mRNA expression compared to DNR group.	[11]
*ACE*	Insertion/deletion (I/D) polymorphism in the ACE gene	DR n = 1039 CTR n = 1185	There was a statistically significant association between tested polymorphism and DR (DD vs. II: OR = 1.73, 95% CI 1.19–2.51; DD + ID vs. II: OR = 1.41, 95% CI 1.16–1.72; DD vs. ID + II: OR = 1.55, 95% CI 1.13–2.12)	[87]
*ACE*	Insertion/deletion (I/D) polymorphism in the ACE gene	DR (DM2) n = 178 DNR n = 206	The D allele of the *ACE* gene is independently associated with DR in Iranian type 2 diabetic patients (OR = 1.831, 95% CI = 1.074–3.124; *p* = 0.026)	[90]
*ACE*	Insertion/deletion (I/D) polymorphism in the ACE gene	NPDR n = 136 PDR n = 94	The DD genotype was more common in the PDR group (*p* < 0.001). Results suggest that the DD genotype increases ACE activity, which in turn increases the risk of proliferation in diabetic retinopathy.	[91]
*ACE*	rs4646994	DR n = 196 DNR n = 200 CTR n = 196	There is no statistically significant difference between DR males and females compared to the corresponding controls. The results were significantly high between DR and DNR genotype frequencies in males. The recessive pattern was found to be significantly associated with DR males (OR = 0.45 [95% CI = 0.20–0.99], *p* < 0.05), while in females they are not significant compared to appropriate controls.	[65]
*ACE*	2350 G/A AA genotype (rs4343)	DNR (DM2) n = 145 DR (DM2) n = 82 CTR n = 90	ACE 2350 G/A distribution genotypes (GG, GA and AA) were as follows: 35.56, 45.55 and 18.89% in the CTR group, 28.57, 46.03 and 25.40% in the group DNR group and 15.85, 46.34 and 37.81% in DR group. No significant differences were confirmed in genotype frequency distribution (*p* = 0.5266) or allele frequency distribution (*p* = 0.2425) between CTR and DNR groups. Distribution of genotype frequencies (*p* = 0.0026) and allele frequencies (*p* = 0.0003) in the DR group showed a significant difference compared to the CTR group.	[77]
*GRB2*	rs9896052	DNR n = 508 DR n = 336	Association with sight-threatening diabetic retinopathy in both the type 2 (OR = 1.50 (CI 95% 1.03–2.18; *p* = 0.035)) and the type 1 (OR = 1.56 (CI 95% 1.02–2.38, *p* = 0.041)).	[93]

DNR—diabetic patients with no retinopathy, DR—diabetic retinopathy, NPDR—non-proliferative diabetic retinopathy, PDR—proliferative diabetic retinopathy, DM2—diabetes mellitus type 2, CTR—control group.

**Table 13:** Polymorphisms of genes related to retinopathy present on chromosome 19.

Gene	Polymorphism or rs Number	No. of Participants	Results	References
*APOE*	e2/e3/e4 polymorphisms	DR (DM1) n = 76; DNR (DM1) n = 96	Neither *APOE* alleles frequencies or *APOE* genotypes frequencies differed between patients with and without diabetic retinopathy	[98]
	e2/e3/e4 polymorphisms	CTR [H] n = 107 DM1 + DM2 n = 141: DM1 n = 46 DM2 n = 95 (who also had DR n = 81)	No evidence of an association between *APOE* alleles and proliferative DR (epsilon2, epsilon3 and epsilon4 in cases: 9, 76, and 15%, and in controls: 5, 88, and 12%, respectively), but the carriers of epsilon2 allele were more frequent among patients with type 2 DM and DR than in controls [cases: 15/95 (15.8%); controls: 7/107 (6.5%); *p* < 0.05].	[97]
	e2/e3/e4 polymorphisms	DNR n = 1144 DR n = 254	*APOE* gene polymorphisms were not associated with diabetic retinopathy in either Caucasians or African Americans. In African Americans, the E2/E4 genotype (n = 6) was associated with increased prevalence of hard exudates (odds ratio [OR] 4.10, 95% confidence interval [CI] 1.30 to 12.90), as was the E2/E3 genotype (n = 9, OR 2.64, 95% CI 1.01 to 6.95). No association between *APOE* genotypes and hard exudates was found in Caucasians.	[12]
	APOE2 rs7412; Arg158Cys); APOE4 (rs429358; Cys112Arg)	CTR [H] n = 2055 DM2 n = 1274	*APOE4* allele revealed an association with retinopathy. Carriers of at least one *APOE4* allele (rs429358) are protected against DM2 related retinopathy (OR [95% CI] = 0.65 [0.42–0.99]. Protection against retinopathy is driven mostly by females (OR [95% CI] = 0.50 [0.25–0.99]); and remains significant (*p* = 0.044) after adjustment for diabetes duration and BMI.	[96]
*ICAM-1*	ICAM-1 469 (K/K, K/E, E/E)	DNR (DM2) [Ret −] n = 50 DR (DM2) [Ret +] n = 81	Allele frequency Ret −: 0.50 for K allele; Ret +: 0.64 for K allele; OR = 1.92 (1.16–3.17). The frequency of *ICAM-1* 469KK genotype and K allele were significantly higher in the patients with DR than in DNR (genotype 42% vs. 20%, χ^2^ = 6.70, *p* = 0.035; allele 66% vs. 50%, χ^2^ = 6.49, *p* = 0.011). These data suggest that the *ICAM-1* 469KK genotype could be a genetic risk factor for retinopathy in Type 2 diabetes mellitus.	[101]

DNR—diabetic patients with no retinopathy, DR—diabetic retinopathy, DM1—diabetes mellitus type 1, DM2—diabetes mellitus type 2, CTR—control group, H—healthy subjects.

**Table 14:** Polymorphisms of genes related to retinopathy present on chromosome 20.

Gene	Polymorphism or rs Number	No. of Participants	Results	References
*PTPN1*	rs3787345	DNR n = 238 DR n = 121	Distribution of genotypes: DR (20.7% CC; 51.2% CT; 28.1% TT); DNR (18.9% CC; 52.1% CT; 29.0% TT); 0 = 0.92	[48]
	rs754118		Distribution of genotypes: DR (34.7% CC; 48.8% CT; 16.5% TT); DNR (34.9% CC; 52.1% CT; 13.0% TT); 0 = 0.64	
*JPH2*	rs761207	DNR n = 575 DR n = 174	1.43-fold (95% CI 1.04–1.98)	[102]
	rs6031415		1.42-fold (95% CI = 1.02–1.97)	

DNR—diabetic patients with no retinopathy, DR—diabetic retinopathy.

**Table 15:** Polymorphisms of genes related to retinopathy present on chromosome 22.

Gene	Polymorphism or rs Number	No. of Participants	Results	References
*TIMP-3*	−899 (T/A) in the promoter region of the gene	PDR n = 113 DM2 n = 158 CTR n = 100	Genotype frequencies of TT/TA/AA were 98.2%/1.8%/0.0% (99.1% A, 0.9% G) for PDR, 96.2%/3.8%/0.0% (98.1% A, 1.9% G) for DM2, and 90.0%/10%/0.0% (95.0% A, 5.0% G) for CTR.	[105]
	−915 (A/G) in the promoter region of the gene		Genotype frequencies of AA/AG/GG genotypes were 47.8%/45.1%/7.1% (70.4% T, 29.6%G) for PDR, 45.0%/43.0%/12.0% (66.5% T, 33.5% G) for DM2, and 40.0%/42.0%/18.0% (61.0% T, 39.0% G) for CTR.	
	−1296 (T/C) in the promoter region of the gene		Genotype frequencies of TT/TC/CC were 47.8%/45.1%/7.1% (70.4% T, 29.6% C) for PDR, 45.0%/43.0%/12.0% (66.5% T, 33.5% C) for DM2, and 40.0%/42.0%/18.0% (61.0% T, 39.0% C) for CTR.	

PDR—proliferative diabetic retinopathy, DM2—diabetes mellitus type 2, CTR—control group.

## Data Availability

No new data were created. All data supporting reported results can be found in analyzed publications.
